# Impact of Oxygen Vacancy on the Photo-Electrical Properties of In_2_O_3_-Based Thin-Film Transistor by Doping Ga

**DOI:** 10.3390/ma12050737

**Published:** 2019-03-04

**Authors:** Kuan-Yu Chen, Chih-Chiang Yang, Yan-Kuin Su, Zi-Hao Wang, Hsin-Chieh Yu

**Affiliations:** 1 Department of Electrical Engineering, Institute of Microelectronics, National Cheng Kung University, Tainan 701, Taiwan; cncer7025@gmail.com; 2 Green Energy Technology Research Center, Department of Electrical Engineering, Kun Shan University, Tainan 710, Taiwan; 3 Advanced Optoelectronic Technology Center, National Cheng Kung University, Tainan 701, Taiwan; kmurder1118@gmail.com (Z.-H.W.); hcyu@nctu.edu.tw (H.-C.Y.); 4 College of Photonics, Institute of Lighting and Energy Photonics, National Chiao Tung University, Tainan 711, Taiwan

**Keywords:** thin-film transistor, oxygen vacancies, co-sputtering

## Abstract

In this study, amorphous indium gallium oxide thin-film transistors (IGO TFTs) were fabricated by co-sputtering. Three samples with different deposition powers of the In_2_O_3_ target, namely, sample A with 50 W deposition power, sample B with 60 W deposition power, and sample C with 70 W deposition power, were investigated. The device performance revealed that oxygen vacancies are strongly dependent on indium content. However, when the deposition power of the In_2_O_3_ target increased, the number of oxygen vacancies, which act as charge carriers to improve the device performance, increased. The best performance was recorded at a threshold voltage of 1.1 V, on-off current ratio of 4.5 × 10^6^, and subthreshold swing of 3.82 V/dec in sample B. Meanwhile, the optical properties of sample B included a responsivity of 0.16 A/W and excellent ultraviolet-to-visible rejection ratio of 8 × 10^4^. IGO TFTs may act as photodetectors according to the results obtained for optical properties.

## 1. Introduction

Amorphous oxide semiconductor thin-film transistors (TFTs) have been widely applied to flat-panel displays, personal digital assistant computers, and smart phones [1]. These transistors are attractive materials as they exhibit electrical characteristics that are superior to those of conventional amorphous silicon TFTs; these characteristics include high carrier mobility, high stability, transparency, and a low-temperature processing requirement [2,3]. Indium oxide (In_2_O_3_)-based binary compounds are excellent materials for TFT devices. These materials are transparent and feature a wide bandgap (~3.2 eV), high electrical conductivity, and optical transparency owing to the native oxygen vacancies that act as donors and indium interstitials. Hence, In_2_O_3_ can act as carrier in multicomponent oxide material systems [4]. In_2_O_3_-based TFTs, as wide bandgap materials, combine electrical and optical functions for photonic integrated circuits, which can serve as photodetectors. Bandgap energy can be tuned by doping other materials, which could be classified as visible-blind photodetectors (λ ≤ 400 nm) and solar-blind photodetectors (λ ≤ 280 nm), although the process depends on the cutoff wavelength [5,6]. 

Among transistor materials, gallium oxide (Ga_2_O_3_) is one of the promising active layers. Ga_2_O_3_ is a wide-bandgap semiconductor with ~4.9 eV; it is an optically transparent material and features poor conductivity [7]. Therefore, Ga_2_O_3_ is the most commonly used component in electronic devices, such as ultraviolet (UV) photodetectors, power electronic devices, light-emitting diodes, and transparent conducting films [8,9,10,11]. However, Ga is a carrier suppressor, which reduces oxygen vacancy in control carrier concentrations in multicomponent oxide TFT devices. The poor conductivity of Ga_2_O_3_ offsets the material’s electrical characteristics. Therefore, doping with indium is often used for effectively improving electrical conductivity [12]. The bond strength of Ga–O is higher than that of In–O, which serves as a carrier that suppresses and improves the stability of components [13]. 

Oxygen vacancies are crystal defects that play a central role in metal oxides and act as donors to provide charge carriers. The components can be optimized by varying the defects [14]. Influencing oxygen vacancy defects through sputtering has been reported by various studies, wherein growth pressure and oxygen flow rate were modulated, and defect concentration and device performance were improved by doping different materials [15,16,17], which can be used in a wide range of applications due to their high deposition rate, easy deposition to metals, alloys, and compounds, enabled control of film composition, high purity, and low cost [18]. Meanwhile, flame detection, oil spill detection, UV astronomy, and semiconductor lithography require UV detection, which can be achieved by using wide-bandgap semiconductors, which are highly transparent and inexpensive and require low-temperature processes [19]. Hence, wide-bandgap semiconductors can be used not only as channel layers for TFT but also as ideal photodetectors [20]. We fabricated indium gallium oxide (IGO) TFTs by using the co-sputtering method, which integrates photodetectors and TFT in measuring the optical and electrical properties of a device and in which measurements depend on the different deposition power requirements of In_2_O_3_ targets. The effects of oxygen vacancies on the characteristics of photodetectors were determined by analyzing thin-film properties using X-ray photoelectron spectroscopy (XPS).

## 2. Device Fabrication and Characterization 

**Figure 1:**
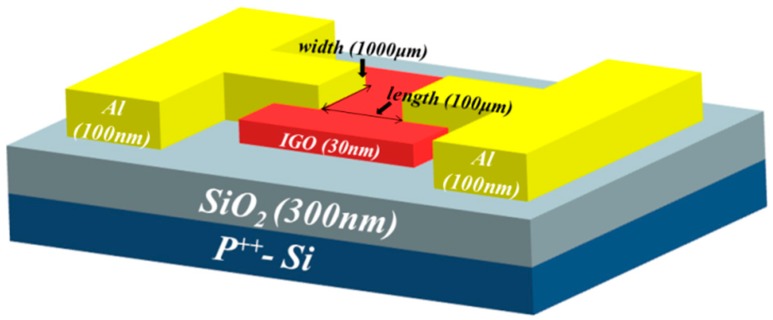
Schematic of IGO TFTs.

Figure 1 shows a schematic of the p-type bottom gate IGO TFT structure used in this study. The 300 nm-thick SiO_2_ dielectric layer was grown on a heavily doped p-type silicon through wet oxidation. Then, the patterned IGO thin film was deposited on the SiO_2_ layer with shadow mask using a radiofrequency (RF) magnetron co-sputtering system (AST, Tainan, Taiwan) at room temperature. The RF powers applied to Ga_2_O_3_ were fixed at 100 W, and the In_2_O_3_ power was set to 50, 60, or 70 W. Argon flow was fixed at 3 sccm under a gas pressure of 5 mTorr and base pressure of approximately 3 × 10^−5^ Torr. For convenience, the RF power condition (Ga_2_O_3_/In_2_O_3_) was set to 100 W/50 W, 100 W/60 W, and 100 W/70 W for samples A, B, and C, respectively. After deposition, the IGO thin films were annealed in an argon environment at 300 °C for 1 h. Finally, the 100 nm patterned Al source and drain electrode were deposited by thermal evaporation. The channel length (L) and width (W) were 100 and 1000 µm, respectively. The thin-film properties were measured by X-ray diffraction (XRD, D8 Discover, Bruker, Billerica, MA, USA), XPS (PHI 5000 VersaProbe, ULVAC, Chigasaki-shi, Japan), atomic force microscopy (AFM, Solver P47, NT-MDT, Moscow, Russia), and UV-visible spectroscopy (U4100, HITACHI, Tokyo, Japan). The electrical characteristics were measured by Agilent B1500 semiconductor device analyzer (Agilent Technologies, Santa Clara, CA, USA) at room temperature and in the dark. The optical properties were measured by Jobin Yvon SPEX system (Kyoto, Japan) with a 150-W Xe arc lamp light source.

## 3. Results and Discussion

**Figure 2:**
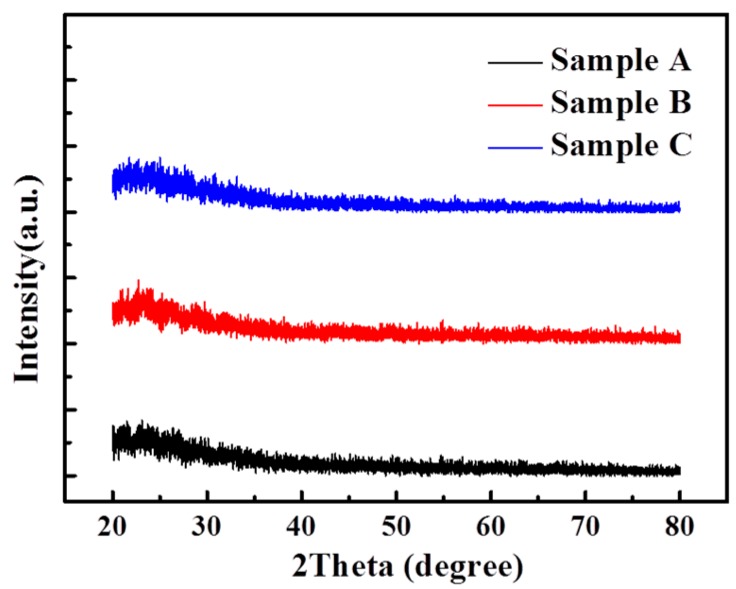
XRD spectra of three IGO thin-film samples exhibited no peak, indicating that all samples were in an amorphous phase.

Figure 2 shows the XRD data of samples A, B, and C. Results showed that no peak was relevant to the three samples, indicating that the samples were in an amorphous phase. 

**Figure 3:**
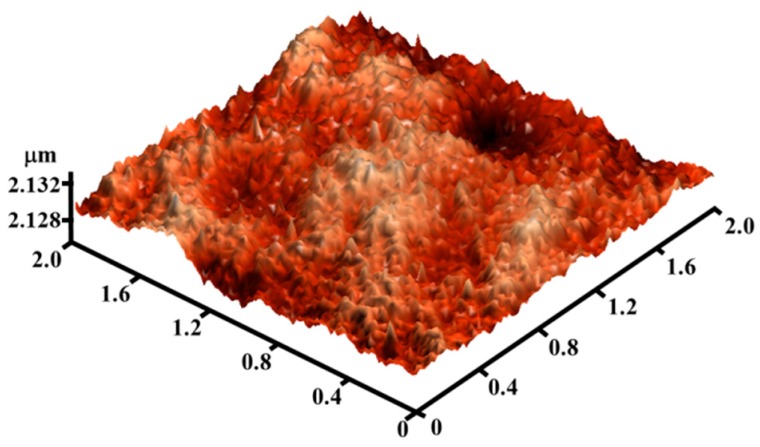
AFM images of dielectric layer exhibiting a smooth surface that could avoid electron trapping at the insulator-channel interface.

**Figure 4:**
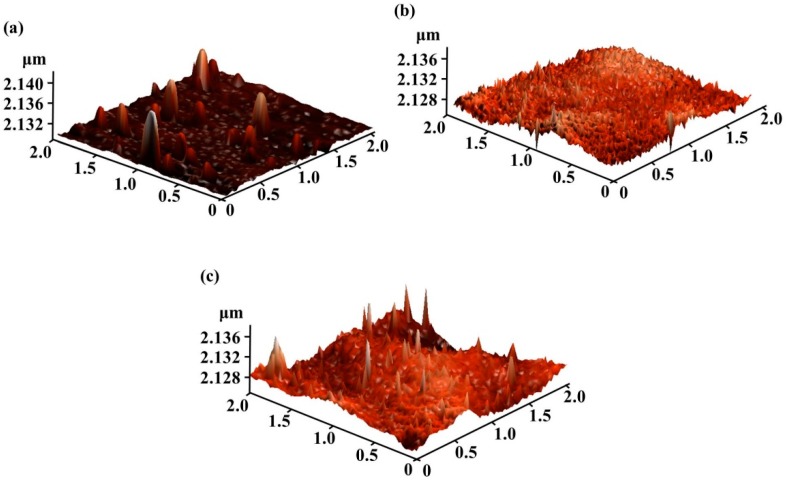
AFM image with RF deposition power of (**a**) sample A: 50W (**b**) sample B: 60W (**c**) sample C: 70W. The Rq values reached 1.207, 1.249, and 1.54, respectively.

The AFM images in Figure 3 show that the root mean square of the insulator layer measured 1.0147 nm. The smooth surface positively influenced the electrical properties and stability; such a condition could avoid electron trapping at the insulator-channel interface [21]. Meanwhile, we performed AFM measurements for samples A, B, and C, shown in Figure 4. The root mean square (Rq) values were 1.207, 1.249, and 1.54 for sample A, B, and C, respectively. The increased roughness could be due to the high deposition rate, which increased along with the deposition power.

**Figure 5:**
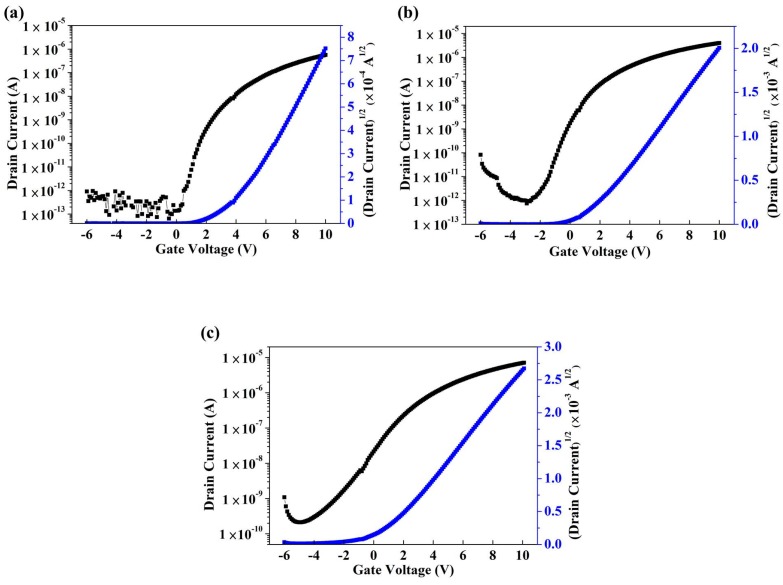
Transfer characteristics of IGO TFTs with different deposition powers of In_2_O_3_. (**a**) Sample A: 50 W, (**b**) sample B: 60 W, and (**c**) sample C: 70 W.

**Figure 6:**
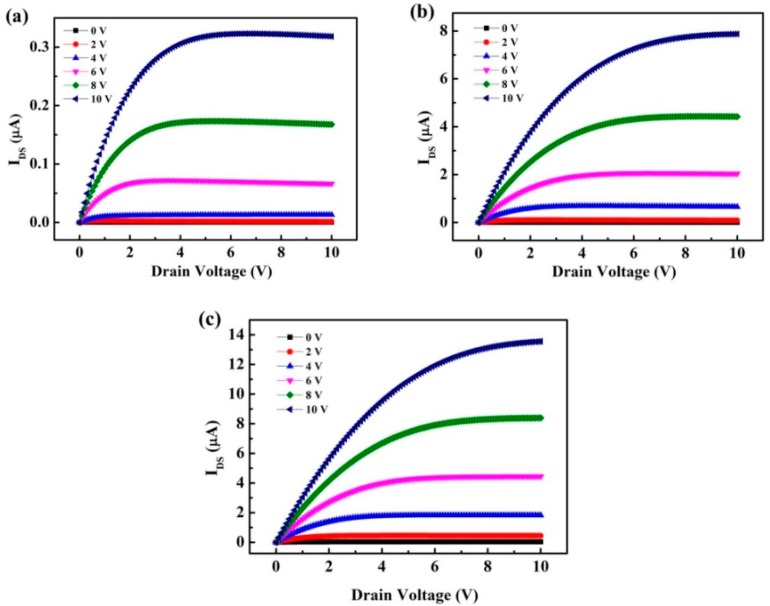
Output characteristics of IGO TFTs with different deposition power of In_2_O_3_. (**a**) Sample A: 50 W, (**b**) sample B: 60 W, and (**c**) sample C: 70 W.

Figure 5 shows the transfer characteristics of IGO TFT for samples A, B and C. Table 1 presents the electrical performance of the threshold voltage (V_th_), on-off current ratio (I_on_/I_off_), subthreshold swing (S.S), and field-effect mobility. The I_DS_ and V_GS_ curves were measured from −6 V to 10 V, and V_DS_ was fixed at 6 V. Field-effect mobility and S.S can be extracted using the following equations:

(1) $$I_{\mathrm{DS}}=\frac{{\mu C}_{\mathrm{OX}}W}{2L}{\left(V_{\mathrm{GS}}-V_{\mathrm{th}}\right)}^{2},$$

(2) $$S=\frac{{\partial V}_{G}}{\partial \left({\mathrm{logI}}_{D}\right)},$$

where C refers to the capacitance of dielectric layer, Vth denotes the threshold voltage, W and L are the channel width and length, respectively. With the increase in indium content, the field-effect mobility increased, and V_th_ exhibited a negative shift as the increased oxygen vacancy concentration increased the carrier concentration. Moreover, the excess oxygen vacancies, which cause an off current, were unsuppressed because of the increase in conductivity of the IGO layer. Carrier concentration was generated by oxygen vacancy formation, and gallium suppressed oxygen vacancy formation owing to the high bond strength between In and O. Thus, when the deposition power of In_2_O_3_ targets increased, the S.S characteristics deteriorated [22]. The optimum performance of IGO TFTs was observed in sample B, which yielded a V_th_ of 1.1 V, S.S of 3.82 V/dec, field-effect mobility of 1.45 cm^2^/Vs, and an I_on_/I_off_ ratio of 4.5 × 10^6^. Figure 6 shows the output characteristics of samples A, B, and C. The I_DS_ and V_DS_ curves ranged from 0 V to 10 V, and Vgs = 0 V–10 V in six steps. The output characteristic curves expressed clear pinch off voltage and current saturation. The saturation drain current was approximately 3.18 × 10^−7^, 7.87 × 10^−6^, and 1.35 × 10^−5^ A at V_GS_ = 10 V for samples A, B, and C, respectively. 

**Figure 7:**
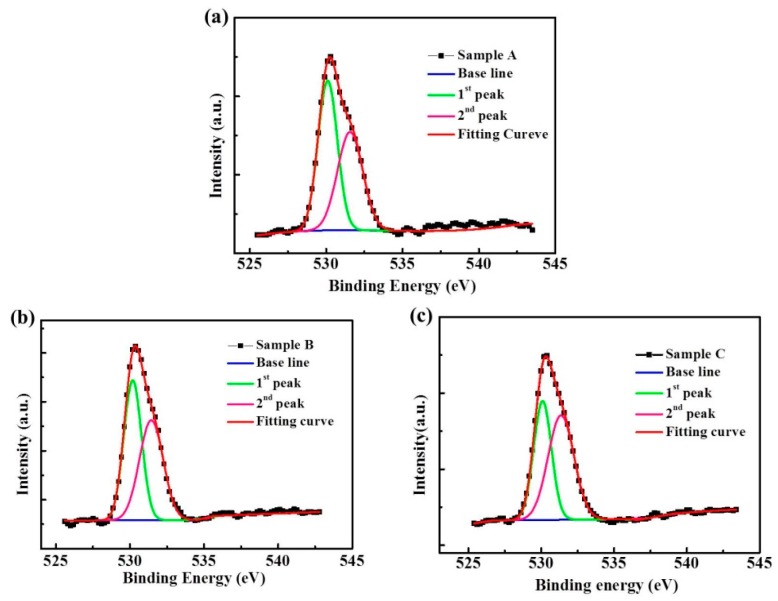
O1s XPS spectra of (**a**) samples A, (**b**) sample B, and (**c**) sample C under various sputtering power.

Figure 7 shows the XPS measurement results, indicating that the O1s XPS spectra of samples A, B, and C feature different deposition powers of In_2_O_3_ target. The O1s spectra were decomposed by Gaussian fitting, which resolved two peaks. The first peak was located at approximately 530 ± 0.2 eV, which is generally attributed to the atomic oxygen of the IGO thin film. The second peak was located at approximately 531.6 eV, which is associated with oxygen vacancies in the IGO thin film. The XPS results indicate that the ratios of oxygen vacancies reached 46.34%, 50.3%, and 56.3% for samples A, B, and C, respectively. These values show that an increase in sputtering power for In_2_O_3_ resulted in higher amounts of oxygen vacancies, that is, from 46% to 56%. Oxygen was easily released because of the low strength of In–O bonds. This finding indicates that In_2_O_3_ content increased as oxygen vacancies increased from 46% to 56%. Oxygen vacancies dominate the conductivity of semiconductors and increase electron concentrations. Thus, oxygen vacancies can act as charge carriers in IGO thin films. Conductivity was improved by increasing the In_2_O_3_ content, thereby indicating the mobility enhancement of IGO TFTs. 

**Figure 8:**
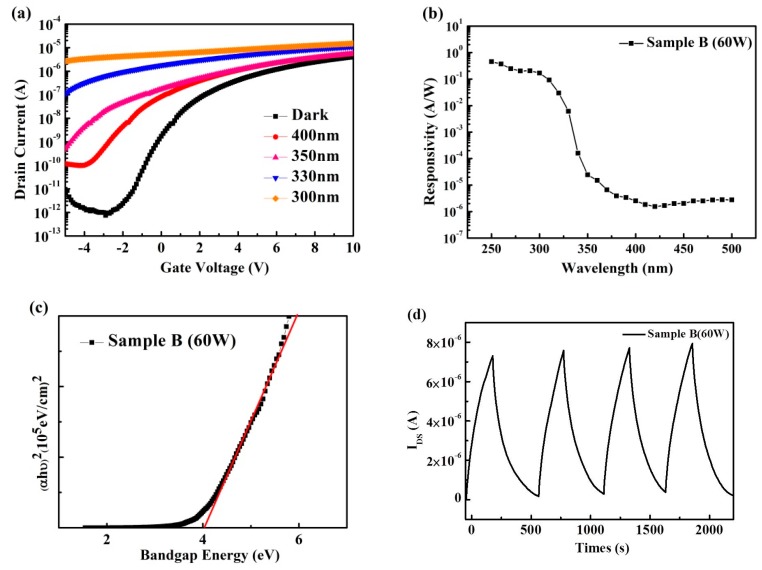
(**a**) Transfer characteristics of IGO TFTs under THE dark region and UV illumination from 400 nm to 300 nm with a V_G_ function from −6 V to 10 V and fixed V_DS_ at 6 V. (**b**) Responsivity of sample B measured in the 350–500 nm wavelength. (**c**) Indirect bandgap values of sample B using Tauc plots and linear fitting. (**d**) Dynamic responses of sample B under UV illumination at 300 nm and with V_G_ = −5 V and V_D_ = 6 V.

Figure 8a shows the transfer characteristics of IGO TFTs under the dark region and UV illumination. Considering that sample B exhibits optimal characteristics due to excessive large number of carriers, sample C featured a larger off current. Hence, the lower I_on_/I_off_ current ratio was 4 × 10^4^; the I_on_/I_off_ current reflects the capacity switching to TFT. On the other hand, a small S.S represents the response speed of TFTs, which require achieving a quick switch behavior. According to the above findings, we defined sample B as the best performing sample. The device was measured under dark condition and under UV illumination from 400 nm to 300 nm. The I_DS_ and V_GS_ curves indicate that the current response of the IGO phototransistor increased after illumination due to photo-generated carrier injection in the channel IGO layer. 

Figure 8b presents the use of a xenon lamp exposure for IGO TFTs, where the responsivity of the device ranged from 500 nm to 250 nm under V_GS_ = −5 V and V_D_s = 6 V. Figure 8c shows the optical bandgap of sample B, and it could be calculated from the Tauc plot expressed as follows:

αhυ = (hυ − Eg),(3)

where α denotes the absorption coefficient, hυ refers to the photo energy, and Eg represents the energy bandgap. Linear fitting of the absorption spectra of sample B was approximately 4 eV for indirect transition, and the cutoff wavelength was approximately 310 nm. In this regard, responsivity could be obtained using the following equation:

(4) $$R=\frac{I_{\mathrm{ph}}}{P_{\mathrm{opt}}},$$

where I_ph_ stands for the output current, and P_opt_ indicates the optical power. The responsivity of the device measured 0.16 A/W, and the UV-to-visible rejection ratio reached 8 × 10^4^. The UV-to-visible rejection ratio was defined as responsivity and measured at 300 nm and divided by the responsivity measured at 450 nm. The highest UV-to-visible rejection ratio signifies that the device was sensitive to UV light. The IGO TFTs show extreme promise as UV photodetectors. Figure 8d shows the dynamic responses of sample B under UV illumination at 300 nm with V_G_ = −5 V and V_D_ = 6 V. The current rapidly increased under illumination and decreased when the light was turned off. The responses measured from sample B were stable and reproducible. The rise time was 24.11 s, and decay time was 21.39 s. In this work, oxygen vacancy concentration changed by In doping, with increased oxygen vacancies leading to improved electric properties of IGO TFTs. as well as, the photo properties show the IGO TFTs have a potential can be used for a UV photodetector applied on flame detection or oil spill detection. 

## 4. Conclusions

In summary, we reported the fabrication of IGO TFTs with different deposition power of In_2_O_3_ target by co-sputtering deposition. The device performance could modulate the content of In_2_O_3_. The oxygen vacancies represented the majority of charge carriers. The number of oxygen vacancies increased with the deposition power of the In_2_O_3_ target. The best device performance was obtained at a deposition power of 60 W (sample B), exhibiting a V_th_ of 1.1 V, I_on_/I_off_ current ratio of 4.5 × 10^6^, field-effect mobility of 1.42 cm^2^/Vs, and S.S of 3.82 V/dec. IGO TFTs can also be used in UV detection. The responsivity of the device was 0.16 A/W, and the UV-to-visible rejection ratio can reach 8 × 10^4^, indicating that the IGO TFTs act as UV photodetectors.

## Figures and Tables

**Table 1:** Electrical performance parameters of IGO TFTs at different deposition power of In_2_O_3_ target.

	I_on_/I_off_ Ratio	S.S (V/dec)	μ_FE_ (cm^2^/Vs)	V_TH_ (V)
Sample A	4.3 × 10^5^	0.526	0.4	3.7
Sample B	4.5 × 10^6^	3.82	1.45	1.1
Sample C	4 × 10^4^	8.3	2.26	0.6
